# Linking Disaster Risk Reduction and Healthcare in Locations with Limited Accessibility: Challenges and Opportunities of Participatory Research

**DOI:** 10.3390/ijerph18010248

**Published:** 2020-12-31

**Authors:** Ilan Kelman, Myles Harris

**Affiliations:** 1 Institute for Global Health, University College London, London WC1N 1EH, UK; 2 Institute for Risk and Disaster Reduction, University College London, London WC1E 6BT, UK; myles.harris.19@ucl.ac.uk; 3 University of Agder, 4630 Kristiansand, Norway

**Keywords:** disaster risk reduction, healthcare, limited accessibility, participatory research

## Abstract

Disaster risk reduction and healthcare support each other, including the mitigation of further harm after illness or injury. These connections are particularly relevant in locations which have permanent or temporary limited accessibility. In these circumstances, people are required to be self-sufficient in providing emergency and long-term healthcare with limited resources. Planning and preparing to mitigate further harm after illness or injury from disasters (disaster risk reduction) must include people living and working in locations with limited accessibility, meaning that participatory research can be used. The challenges and opportunities of enacting participatory research in such contexts have not been thoroughly examined. The research question of this paper is therefore, “What challenges and opportunities occur when participatory research links disaster risk reduction and healthcare to mitigate illness and injury in locations with limited accessibility?” To answer this research question, the method used is a qualitative evidence synthesis, combined with an overview paper approach. Two principal themes of challenges and opportunities are examined: defining the data and collecting the data. The themes are explored in theory and then through contextual examples. The conclusion is that an overarching challenge is divergent goals of research and actions that, when recognized, lead to opportunities for improved connections between disaster risk reduction and healthcare.

## 1. Introduction

Healthcare and disaster risk reduction (DRR) have always been closely connected given that they support each other [1,2,3]. To frame the connections and to provide the scope for this paper’s mandate in Section 1.2, detailed discussion of definitions is provided first in Section 1.1.

### 1.1. Definitions

“Disaster” has no fixed or universally agreed definition but is typically summarized as being a situation requiring outside support in order to deal with it [4,5,6,7]. Thus, “disaster risk” refers to the potential for adverse outcomes due to a disaster, often focusing on casualties (deaths and injuries), infrastructure damage, livelihood interruption, and disruption to day-to-day activities [7,8,9]. That is, disaster risk is the potential for the need for outside assistance, while a disaster is when it actually happens.

These concepts lead to the fundamental point of “DRR” being defined as tackling the root causes of disasters and disaster risk, namely vulnerabilities, with a disaster typically manifesting when those vulnerabilities intersect with hazards [10,11,12]. Examples of hazards are floods, earthquakes, and wildfires, yet the disaster is not caused by these natural processes (even where modified or triggered by human activity). Instead, the disaster emerges from choices to create and allow vulnerabilities—such as through wealth inequities, discrimination against populations, and failing to provide education—so that people do not have the information, resources, or empowerment to prevent harm from the hazards [10,11,12]. Thus, the term “natural disaster” is a misnomer while preventing and responding to disasters means addressing vulnerabilities, irrespective of what nature does [13,14,15].

Healthcare is defined as achieving and maintaining a state of good health and wellbeing, relative to each individual person; thus, health and healthcare are substantially socially determined [16,17,18]. Achieving the state which is the definition of “health” means continual, proactive approaches for individual and collective physical and psycho-social health needs as an ongoing process, including when people are living with long-term health conditions. In other words, caring for and promoting one’s health continually becomes healthcare, not just seeking professional medical attention when injured or ill.

As per the literature cited above, the definitions of DRR and healthcare are evidence that they are long-term processes, focusing on mitigating, reducing, preventing, and preparing for harm or damage, which must incorporate not worsening situations once harm or damage have manifested. Nonetheless, as the literature above notes, treatment is only part of healthcare, just as disaster response is only one part of dealing with disasters, although definitions can separate DRR and disaster response to a large extent, despite their interplays as noted in the literature cited above. Thus, the definitions of DRR and healthcare complement one another because they have the same goal of promoting good health and reducing poor health.

Implementing DRR and healthcare in tandem, including the aspects of mitigating further harm, damage, illness, or injury, applies to locations with temporary or permanent limited accessibility. No standardized or consistent definition exists of “limited accessibility”, especially since much is subjective and a location’s accessibility can change rapidly (Table 1). That is, physical remoteness, isolation, or perceived or actual marginalization is not necessarily a condition for limited accessibility, as shown in Table 1. Actual marginalization, though, can still play a role, with many documented examples of healthcare, DRR, vulnerability reduction, and disaster response disfavoring populations which are marginalized due to lower socioeconomic status [19,20].

Limited accessibility poses challenges for DRR and healthcare because mitigating further harm or damage after it has occurred might need to continue for a prolonged time until the patient’s healthcare needs are met, such as through external support or recovery [21,22]. The nature of a location with permanent or temporary limited accessibility affects the provision of healthcare, which can include patient needs and resources available regarding treatment such as to mitigate further problems.

Detailing some examples from Table 1 for permanently limited accessibility, many island [23] and mountain [24] locations provide DRR and healthcare under circumstances of limited transportation, few resources, and sustained self-reliance [25,26,27]. Similar characteristics can appear temporarily after a disaster, if transportation routes or communication lines are cut, or if supply chains are interrupted. For example, emergency planning for New Zealand—an island and mountain country—after a major earthquake in the national capital, Wellington, assumes that accessibility and supplies will be limited for days—in some cases, months—after the shaking stops [28]. Earthquake disasters in Haiti (an island country) in 2010 and China in 2008 (in a mountainous region) devastated services, supplies, and supply chains, impeding inbound people and materials [29].

In such circumstances, mitigating further harm and damage is needed for people living with long-term conditions (chronic healthcare) and those who need emergency assistance (acute healthcare) [30,31,32,33]. For this mitigation to be fully effective, key people involved (those who are directly affected) need to be included in producing and testing recommendations and actions, due to the self-reliance necessary for providing DRR and healthcare in locations with limited accessibility [34]. Research methods for investigating this topic in a systematic, verifiable, and robust manner are needed, while including the people affected. These concepts lead, therefore, to participatory research, ensuring that people are directly involved in the research process [35,36,37], which can be qualitative, quantitative, or mixed methods [38,39].

Participatory research within healthcare contexts can be described as collaborative research between researchers, healthcare users, and healthcare professionals (covering, for instance, scientists, research support staff, patients, service users, suppliers, and healthcare providers) [40,41]. Methodologically, participatory research can be quantitative, qualitative, or mixed methods [38,39]. One method is not better than another. Instead, the choice of which method to use to conduct participatory research should be based on the appropriateness for answering a well-defined research question or questions. Examples of participatory research for healthcare are women’s groups for reducing material and infant mortality [42] and developing mobile phone games to improve health awareness [43]. Improving such approaches is suggested as being needed to reduce HIV infection and intimate partner violence in Southern Africa [44]. Participatory research for healthcare means that all parties are involved in the research, aiming for positive health-related outcomes. Participatory research with a qualitative methodology tends to align more closely to healthcare research covering all aspects of health, including physical, psycho-social, spiritual, occupational, and environmental health. Participatory research, however, still can and often should be informed by quantitative methods or mixed methods, providing that the methods suit the answering of the well-defined research question(s).

Participatory research for DRR contexts is similar, with researchers collaborating with people affected by and dealing with disasters and disaster risk [45,46]. Examples are dealing with flooding in Mozambique [47] and self-reflection on developing warning systems in Brazil [48]. These case studies across decades and continents demonstrate how the people experiencing disaster risk have plenty to contribute to DRR work, thereby ensuring the best chance of success. Participatory research is a method that can be used to include peoples’ experiences, which enriches research findings and, moreover, ensures that the findings are bespoke to a specific context. Participatory research from a DRR perspective often adopts qualitative methodologies, but the possibility and need remain for using quantitative methods or mixed methods when appropriate for the research question(s).

The importance of participatory research when science and its implications can affect people’s lives is shown by the long-standing philosophies that science is not a linear, impartial, objective, process in which neutral knowledge is easily packaged for simple communication to people affected as a one-way route from “expert” to “user” [49,50,51]. In contrast, human judgements, subjectivities, and values imbue all steps of the scientific process, meaning that scientists’ views might differ from the perspectives of the people whom the science is about or who are affected by the science [52]. The issue is not about who is “right” and who is “wrong”, since all knowledge forms have limitations [53,54]. Instead, it is about collaboration, to cross-check material and relevance, aiming for as complete and as useful an understanding as possible, so that the people affected by DRR and healthcare gain from the research. Hence, participation in the entire research process is important and such collaboration enhances the generalizability of research findings, as demonstrated by the examples in the references given in this section.

The roles of academics and of academia within participatory research have been called into question [55,56] along with the risks and alleged inconsistencies which emerge due to the realities of dealing with people during fieldwork [57]. Based on these cautions, both healthcare and disaster researchers query how participatory such research necessarily is and especially where improvements ought to be enacted, because they document similar challenges in applying it for healthcare, DRR, their links, and their broader contexts [58,59,60]. This work and the lessons emerging from it, especially where problems arise, provide a baseline for adapting the knowledge to under-researched situations such as locations with limited accessibility and ensuring that the known pitfalls are avoided [39,61].

### 1.2. Research Question

To improve the understanding of this topic, and to provide methodological guidance for researchers and practitioners, this paper investigates the question, “What challenges and opportunities occur when participatory research links DRR and healthcare to mitigate further illness and injury in locations with limited accessibility?” The aim is to provide a foundation of literature-based recommendations for ensuring that participatory research is enacted effectively for linking DRR and healthcare to mitigate illness and injury in locations with limited accessibility.

To answer this research question, the method used is a qualitative evidence synthesis, combined with an overview paper approach [62]. This method is similar to a literature review (not a systematic literature review, but a broad examination of the literature with a narrative exploration), although the method used here is more appropriate for this paper due to its focus on surveying the literature and describing the characteristics and impact on practice [62] The qualitative evidence synthesis is a critical review of evidence about a defined theme, aiming to formulate an evidence-based description of that theme for dissemination. The evidence comes from examples and sources, selected by using the overview paper approach of purposive sampling (which some label as a subset of selective sampling). [36] (p. 1670) support purposive sampling in participatory research for “reliability and representativeness” (which should also be interpreted to encompass validity) while [63] (p. 124) note its importance for “identification of differing views and perspectives”, corroborating [64]’s explanation of how the method is appropriate in health for obtaining a variety of examples, exactly as per the aim here. The point of this method is not to be comprehensive or systematic, but to ensure a variety and balance of examples, providing an overview of the topic [62], as has been applied to aspects of connecting humanitarian aid and DRR [65]. In other words, the findings of this paper are useful for informing participatory research, using any method or combination of methods, in areas with limited accessibility. The method effectively collates and critiques the evidence on the selected theme for indicating relevant issues and research gaps, enabling the findings to be generalizable and applicable to practice.

The next section summarizes two known, specific, data-related challenges of participatory research methodologies as applied to this paper’s topic. Section 3 uses case studies to illustrate these specific challenges, and how they might be overcome, in practice. Section 4 syntheses the material and provides recommendations through exploring limitations and further interpretations of the evidence. The conclusions describe the main contributions of this paper and indicate some gaps to be filled.

## 2. Data-Related Challenges

Plenty of research on linking DRR and healthcare in locations with limited accessibility can be conducted conceptually (such as this paper), for modeling [66], in laboratory conditions [67], and in controlled field conditions [43]. Other research approaches involve examining real situations in real time [68] or post-hoc analyses [69]. Challenges emerge in using participatory research to investigate these topics when defining the data (Section 2.1) and when collecting the data (Section 2.2), which are then summarized in Section 2.3. The morals of conducting such research, as well as how institutional and national ethical approval does not necessarily resolve all the concerns, are well documented [70,71,72,73]. The discussion in this section assumes that no research should be carried out without the appropriate approvals—including data protection, background checks, informed consent, ethics approval, and risk assessments—in order to focus here on the pragmatic challenges of what the data are and how they would be collected, with the ethics and risks already presumed to be acceptable.

In science, data are, at basis, a collective of information, which might be numbers, words, symbols, images, other visuals, sounds, other sensory stimuli, or other representations of knowledge or observations. Data can be compiled, compared, abstracted, and analyzed, leading to fundamental theories of data involving aspects such as scaling, measuring, categorizing, and connecting the information [74,75,76]. These decisions and interpretations lead to bias [50], meaning that data cannot be taken as neutral or objective, with some authors even questioning what data would be legitimate [57]. As [77] (p. 45) notes, “Data is in the eye of the beholder”. Hence, research methodology transparency enables the reviewer to critique the results or findings before applying them to practice [78].

### 2.1. Defining the Data

A frequent expectation in research is defining the case study and unit of analysis [79]. A case study is a method used to research something in-depth within its bounded context and a unit of analysis is the object or process of enquiry about which most data are collected and analysed [79]. In dealing with DRR and healthcare in locations with limited accessibility, neither a case study nor a unit of analysis is always neatly definable or clearly delineated, especially because the incidents being examined do not always occur regularly or predictably. The unit of analysis might be the specific patient, the health situation, such as a snake bite, or the circumstances leading to the health situation, such as one volcanic eruption. The case study might be similar, such as a specific volcanic eruption or terrorist act, or it might be a wider temporal or spatial scope, such as overwintering in Antarctica or collecting reptiles in a location with limited accessibility.

Because of the rarity and lack of commonality of many of the situations investigated (see Section 3), people’s experiences are important, especially the words of patients and medical staff, meaning that narratives become essential data, especially for articulating each situation’s circumstances. These narratives produce legitimate and sometimes representative data, expressed as auto-ethnography [68], participant–observer [80], and first-hand accounts [81]. For research publications, few problems emerge, as these are all appropriate and accepted methods, particularly when considering the need for data to be representative and applicable to a context or area of practice [82]. For developing manuals, training, instructions, and guidance, transferability and generalization pose difficulties due to the need to adapt to specific circumstances as well as limited verifiability and repeatability. When conducting research on patients’ needs [83] or perceptions [84] in locations with limited accessibility, what the healthcare actually was and its effectiveness represent different research questions. When determining the healthcare to provide and being able to assess its effectiveness, anecdotal or ad hoc material is difficult to use due to the dynamic nature of locations with limited accessibility [30].

Instead, a large, comparable sample size would be preferred. By the very nature of the problem defined (participatory research for linking DRR and healthcare in locations with limited accessibility to mitigate further harm or damage), specific examples are not always available and sample sizes tend to be small due to lower population sizes which are frequently present in areas with limited accessibility. The island of Tristan da Cunha in the South Atlantic and many settlements on small islands in Northern Kalaallit Nunaat (Greenland) lack both an airport and a large harbor, so bringing in medical equipment and health personnel is not easy. The differences are nonetheless substantial. Many of the settlements in Kalaallit Nunaat (Greenland) are accessible by helicopter, depending on the weather, whereas Tristan da Cunha is out of range of land-based helicopters, and the settlements in Kalaallit Nunaat must contend with far more wintry conditions than Tristan da Cunha. Whether or not islands inevitably have commonalities is debated [23,85] and there are certainly some similarities between the two examples provided here. Using them as comparable units of analysis simply to increase the sample size would be difficult to defend, as would island examples as the case study. Even just considering the settlements in Northern Kalaallit Nunaat would be problematic for comparison, because their accessibility varies substantially across the modes of access of helicopter, airplane, boat, dogsled, snowmobile, and skiing as well as the people who are seeking access to or from the locations [86]. Furthermore, seasonal conditions increase the complexity of DRR and healthcare provision. The large number of influencing factors often inhibits robust comparisons across time, geography, or circumstance.

A sample size of one is accepted in some research [68,87] and having a single, stated-to-be unique case study for a study is publishable for both DRR and healthcare research [88,89]. The findings are admissible research which stands in its own right, while transparently discussing the limitations, as must be the case for all research. Extracting lessons to be applied elsewhere and providing practical outcomes from such work is less certain. However, if everything is contextual or if every case study is unique, what techniques are needed to move from science to policy, practice, and action with confidence and is this a useful goal [90,91]?

One advantage of small units of analysis is that a sample might reach 100%, or close to 100%, of the population in the location [92]; hence, the findings are usually representative of the location’s population. The research might thus be publishable, but not useable for predictive or explanatory models, because the sample is size is too small or too specific to its context. A small sample size can thus be a challenge for participatory research.

### 2.2. Collecting the Data

In situations where the data are acceptably defined and can be used for both research and application, difficulties could arise in collecting the data because providing healthcare and enacting DRR are legitimately more important than science; that is, actually mitigating further injury or illness should supersede researching it. Irrespective of formal institutional or national research ethics approval, being involved in disaster-related activities at the same time as researching the topic in real time has always required robust ethical and operational considerations [72,93].

Auto-ethnographic methods and post-hoc writing up and analysis of observations can overcome the issues noted in the previous paragraph to some extent [93,94]. Then, the question arises on how to ensure that actions are not adversely influenced by the knowledge that they will be deconstructed, analyzed, critiqued, and published afterwards. In other words, the potential introduction of subject bias requires transparent discussion, in that the knowledge that research is being conducted on operational activities could influence how those activities are enacted [72] by making different decisions about healthcare and communication. This concern is well known for action research, as noted for post-genocide Rwanda [95] and incidences have been documented of rescue protocols being altered due to media presence, labeled as Rescuetainment [96]. Extra consideration of practice is not inevitably bad, as it can enrich both research and operations, even if different ethics approval approaches are needed [97]. The issue of making comparative analyses, however, emerges again.

All healthcare, including treatment, is dynamic because it is person-centered, responding to the individuality of and changes within the patient’s physical and psychological condition [98,99]. Consistency is rarely possible or desirable. In locations with limited accessibility, procedures vary further according to the availability of personnel, expertise, equipment, supplies, and communications [100]. While the first patient with a specific condition might receive an MRI scan or a ventilator, the tenth might not because the MRI contrast agent has run out or because all working ventilators are in use. If a specialist treating a queue of patients becomes exhausted or injured, then treatment regimes must change. Thus, collecting data about DRR and healthcare in locations with limited accessibility is an opportunity to identify, analyze, and synthesize the findings, exploring the interrelation of these two disciplines that will inform future DRR and healthcare in those environments.

Additionally, even for auto-ethnography and first-hand accounts, interacting with and interviewing others could be important for the research to determine exactly why and how decisions were made and what alternatives were considered. Doing so during a rescue, medical evacuation (also known as medevac), or rest periods could violate ethical principles in research and operations, meaning that valuable real-time data on perceptions, decision-making, and reasoning are lost. Similarly, not all patients can be interviewed consistently due to the effects of pain, shock, pharmacological side effects, or focus on other matters, thus being distracted from the research process. Finally, as is applicable for both disaster and healthcare research, “The fact that those who did not survive cannot tell their stories automatically makes one gap in data on survival behavior” [93] (p. 4). Some methods exist and have occasionally been applied to reconstruct aspects of such decision-making for flash flood evacuation [101], voice messages left during the 11 September 2001 terrorist attacks by people on hijacked planes and above the World Trade Center impact sites [102], and tunnel fires [103]. Not being able to include fully the perspectives of people who have died in disasters in locations with limited accessibility is therefore a challenge for participatory research in these contexts.

No data and no research process can ever be perfect [50], especially for fieldwork where controlling conditions and all influencing factors might not be feasible or desirable, such as for the health impacts of temporary shelters in cold environments [104] and other disaster research [93]. The methods used throughout the research process must be appropriate to the research question or topic to ensure that the findings or results are robust [105]. A balance must be sought between research on mitigating further injury or illness and the research itself not causing further harm or damage, or interfering with any of the participants. Participatory research is an opportunity to conduct inclusive and collaborative research for people in locations with limited accessibility, who require bespoke DRR strategies and have specific healthcare needs. Furthermore, participatory research provides an opportunity to collect meaningful data for the people in these locations, thereby potentially having a more significant impact for them in comparison to research with alternative methods.

### 2.3. Summary of Data-Related Challenges

Table 2 summarizes this section by providing key categories of the identified data-related challenges.

## 3. Examples

This section of the paper describes examples of linking DRR and healthcare in locations with limited accessibility for mitigating further illness and injury to indicate the challenges and opportunities for participatory research, which are then elaborated in Section 4. These are effectively operational scenarios in which the difficulties described in Section 2 might arise, divided into individual casualties (Section 3.1) and multiple casualties (Section 3.2), followed by a summary based on Table 2 (Section 3.3). They were chosen and classified through using the overview paper approach [62].

Because the cases are specifically selected to present a variety and because the situations described by this paper tend to have their own uniqueness, not all of them can have explicit participatory research. In some cases, the key is articulating how participatory research could potentially (have) contributed to the situation and the possible considerations which then might have arisen. Given the basic ethos within DRR and healthcare that prevention is better than cure, thinking ahead of challenging situations is fundamental; thus, the identified methods for answering this paper’s research question are appropriate. The absence of participatory research within a specific situation of limited accessibility should never preclude analyzing hypotheticals and thinking about what could have been done.

### 3.1. Individual Casualties

Individual cases have been documented in narratives and literature illustrating some of the specific challenges which need to be considered for mitigating further injury or illness in locations with limited accessibility in order to link DRR and healthcare. They are not necessarily wider disasters in the strictest definitions of “disaster”, which are debated and flexible [5,6], but for the group involved, the situation was disastrous and they are indicative of scenarios which could happen at a wider scale.

A biology expedition in remote Burma in September 2001 had organized two doctors and a radiophone, but it turned out that neither the personnel nor equipment were available, so the expedition continued without them [81]. When the team was almost 13 km from the nearest radio, the lead scientist was bitten by a krait, a poisonous snake for which the venom induces paralysis for up to two days. The team’s efforts at artificial respiration and the eventual arrival of medical personnel were not enough to save the scientist, because neither an air evacuation nor a proper respirator could be arranged. Snakebite deaths in Burma have long been common [106] and the lack of health services combined with limited accessibility in many locations demonstrates issues of emergency treatment of venomous snakebites [107]. This case study involves one person with illness and injury and the attempts to mitigate it, making it an example of a small-scale disaster, during which prolonged healthcare provision was required in an area with limited accessibility. Participatory research could potentially have been conducted, once emergency care had been provided; however, there are many influencing factors to be considered in this example.

Given the urgency to keep the bitten man alive through continual artificial respiration and possibly cardio-pulmonary resuscitation (CPR), which might have needed to be continued for two days, thereby exhausting the people involved, conducting any form of real-time participatory research would have been difficult and would raise ethical contraindications. Post-hoc analysis might be skewed by guilt and grief, as illustrated in [81]’s description, especially regarding prevention rather than mitigation. From a participatory research perspective, although a small sample size cannot represent everyone experiencing a snakebite, the findings are applicable to the context and environment, covering both the time and place. Furthermore, transparency of methodology could identify transferable lessons for similar research in other areas with limited accessibility. This case mirrors other disasters, such as floods, during which snakebites are common [108], raising the specter of how to research the treatment of multiple recipients of venomous snakebites to prevent further illness or injury amidst multiple other casualties.

Antarctica is a continent with limited accessibility. It is governed by the Antarctic Treaty System (ATS), covering all locations south of 60° S and setting aside the region for science, with researchers requiring permits and health checks to work there. Antarctica is an example of how conditions vary substantially and rapidly change in the same location, so comparing case studies of DRR and healthcare may have major difficulties. During the summer, the level of accessibility is reasonable, although the environment remains challenging due to distances, elevation, temperature, storms, and ice; thus, DRR and healthcare logistics remain expensive and unreliable. Many research stations become entirely isolated during the winter, because the environmental conditions preclude transportation in and out, as shown by a trio of medical emergencies.

In 1960–1961, a Soviet team built a new inland research station to spend 1961′s winter there. In April, the team’s sole doctor diagnosed himself with acute appendicitis. After trying other treatments unsuccessfully, the only remaining possibility was to remove his appendix, which he did himself in an operation called an auto-appendicectomy, after which he made a full recovery [109]. The situation is explained as being “in the wilderness, out of hospital settings, with no possibility of outside help, and without any other medical professional around” [109] (p. 1422), which is the epitome of the need for prolonged healthcare to mitigate further injury or illness in locations with limited accessibility. This instance is another example of a participatory research study with a small sample size that provided an opportunity to investigate the provision of healthcare in an area with limited accessibility. Although circumstances such as these may appear rare, there are other documented examples.

Another doctor who had to self-treat during the Antarctic winter was the only physician at the South Pole station in 1999. She discovered a lump in her breast, did a biopsy, and found that it was cancerous. Supplies were dropped by air for self-treatment until it was possible to evacuate her in October during a dangerous landing and takeoff [110]. This case study has been used as an analogy for planning for similar scenarios during space flight [111], which is an example of how the findings of a participatory research study can inform practice in other locations with limited accessibility.

In both these examples, the patient was able to reflect on their situation and make case notes, providing material and scope for real-time participatory research. While neither appendicitis nor breast cancer are unique, different healthcare regimes can occur for different patients and even the same healthcare approach is typically tweaked for each patient. The expected time to evacuation can make a difference. The patient with appendicitis knew that there was no early route out of Antarctica and that inaction likely meant death. The patient with breast cancer knew that efforts were being made for the earliest possible evacuation flight, although with a large window regarding when it would be feasible, as well as the fact that some of form of treatment, substantially reducing the chance of dying, was feasible while awaiting evacuation.

The third Antarctic incident took place in July 2003 when a snorkeling scientist was attacked and dragged underwater by a leopard seal. Despite rapid rescue of the scientist and a long resuscitation attempt, she became the first known human fatality from a leopard seal attack [112]. If she had been revived, recovery after CPR could have been a long process which would have proceeded in the Antarctic winter until evacuation would have been possible. During the rescue and recovery efforts, conducting participatory research would have had significant ethical and operational implications. If the scientist had been revived, then real-time participatory research into mitigating further illness or injury could have been conducted by and with the patient, the medical staff, and the research team. This example represents a challenge of participatory research, within the context of providing healthcare to individuals in an area with limited accessibility.

### 3.2. Multiple Casualties

Challenges similar to those in Section 3.1 are evident beyond a patient sample size of one, namely in multi-casualty situations.

On 22 July 2011, a terrorist detonated a bomb in Central Oslo, killing eight people, drove approximately one hour to a dock, and boarded a ferry to the island of Utøya, where 564 people, mainly youth from the governing Labour Party (Arbeiderpartiet), were holding a summer camp. The terrorist started shooting and then hunted down people trying to flee, including firing at those in the water swimming away. By the time he gave himself up to be arrested, 69 were dead and around 110 injured. Limited access to the island and to the nearest mainland area by land, water, and air led to difficulties in staging and treating casualties [113], including providing advanced life support and in recovering casualties while an active shooter (or more than one) was known or thought be at large. A participatory research study that includes the healthcare providers in this example would provide valuable findings that could inform DRR for terrorism-related disasters requiring healthcare provision in areas with limited accessibility.

Whakaari (White Island) is a privately owned island in the Bay of Plenty, 48 km from the east coast of New Zealand. It is an active volcano and was a popular tourist attraction, given the opportunity to take a boat trip and then to walk inside a volcano’s crater. On 9 December 2019, the volcano erupted while 47 people were on the island, with 34 being rescued, all of whom were injured, including many with severe burn and acid damage. After the immediate rescue operation, 13 were declared missing or dead, with the death toll eventually rising to a total of 22, although two bodies were never recovered.

Limited accessibility arose at three levels. First, Whakaari’s distance from the mainland delayed the availability of advanced life support while the volcano’s eruption impeded rescue and immediate treatment. Second, capacity at the nearest hospital in Whakatäne was stretched [114], with an intensive care nurse noting, “I have seen some terrible injuries and burns—but never so many casualties at one time,” [115] (p. 10), so some patients had to be transported elsewhere immediately. Third, New Zealand is a small country and struggled to treat and rehabilitate so many patients with burn injuries [116] due to the amount of skin grafts and length of healthcare required at around the same time. Many of the injured were soon repatriated out of New Zealand, some of whom died in their home country. The Whakaari volcano eruption exemplifies how healthcare services can be rapidly overwhelmed in the response to disaster due to limited capacity and resources. Participatory research would provide an opportunity to investigate the provision of acute and long-term healthcare for people in a location with limited accessibility, and in this example, there is a larger sample size, which could result in more representative findings.

For both the islands, an abundance of data exists with a good sample size, but conducting real-time participatory research on the efforts to mitigate further harm or damage in the areas of limited accessibility would not be possible due to the lack of personnel and the possibility of interfering with healthcare. Hence, a challenge of participatory research is balancing healthcare provision and conducting research. The first priority of any researcher present should be providing first aid, including psychological support and reassurance to survivors, rather than trying to collect data, or else the research becomes unethical. Once the patients are transported to larger centers, such as Oslo for Utøya and Auckland for Whakaari, prospects exist for participatory research with the patients and the medical staff into mitigating further illness and injury, which was completed for Utøya [117] and which seems to be ongoing for Whakaari [116]. Thus, the timing of participatory research is an important consideration for the researchers and people involved.

On the morning of 25 April 2015, an earthquake struck North-Central Nepal, killing over 9000 people, including at least 15 and up to 21 at Everest Base Camp, in mountaineering’s most lethal disaster so far. The deaths came when an avalanche triggered by the earthquake created a massive pressure wave flattening tents. Three more people died trying to find a route back for climbers trapped above Base Camp. Dozens were injured at Base Camp, the main medical tent was destroyed, and at least one doctor was severely hurt.

The rescue, triage, treatment, and evacuation were described by those there as being ad hoc and improvised due to the challenges of limited accessibility [69], which is an example of post-hoc analysis, also describing local participation, such as in places where the evacuation aircraft stopped en route to Kathmandu. The paper’s authors involved in the treatment were not conducting participatory research at the time of the disaster, instead waiting until afterwards to collate, synthesize, and analyze their data.

War zones are locations with limited accessibility, with the need to link DRR and healthcare for mitigating further illness and injury, but they have difficult participatory research conditions [118,119]. War zones have limited accessibility due to the high risk of injury or death as a result of conflict, or reduced accessibility fueled by political sensitivity or censorship [120]. Historical studies [121,122] contribute, as do descriptive [123] and observational [124] data collection. The journal *Military Medicine* has been published since 1940 and lists 35 papers with the word “participatory” in the title, abstract, or full text, only one of which was about combat medicine [125]. Nonetheless, the literature on participatory research in war zones is large, including auto-ethnographies [68] and volunteers trying an intervention [126], with the work covering a wide range of issues on data, ethics, operations, transferability, and researcher roles. War and conflict case studies hence provide evidence that using auto-ethnographic or participant–observer methods can provide an opportunity to enable valuable (and sometimes the only) contributions to DRR and healthcare research in such areas with limited accessibility.

### 3.3. Linking to the Data-Related Challenges

Table 3 summarizes the data-related challenges, correlated with information in Table 2, which apply to each example in Section 3.1 and Section 3.2. In each instance, the vulnerabilities of the individuals and collectives involved match up with the basic disaster risk theory presented in Section 1.1 in that known vulnerabilities led to the adverse outcomes. For the three Antarctic cases, as well as Burma, the vulnerabilities and risks were well known and were assessed before, but the actions went forward anyway on the basis of accepting the risks and taking as many mitigative actions as feasible. These actions led to a successful outcome in two of these four cases. For Utøya, the official report documented numerous failings and missed opportunities to stop the terrorism, which was a known threat [127].

## 4. Synthesis and Discussion of Challenges and Opportunities

Section 3 demonstrates the diversity of ways in which DRR and healthcare are linked through mitigating further illness or injury in areas with limited accessibility, especially for participatory research, as well as highlighting the dynamic nature of these situations. This section provides further synthesis and discussion on the challenges and opportunities in the context of participatory research.

Davenport et al. [128] researched the effects of exercise at a high altitude on a pregnant woman (sample size of one) who chose to continue employment as a trekking guide in the Nepalese Himalayas. Her physical and mental health was closely monitored during an expedition from Namche Bazar, Nepal (3440 m in elevation) to Everest Base Camp (at approximately 5300 m in elevation) and back again. The participant was both the case study and the unit of analysis—although the case study might also be physical activity during pregnancy at high altitudes—but her safety was the predominant concern for the researchers. Data collection was still managed, although as a secondary priority. Ethical approval was applied for and received before the study started. It was an opportunity which was grasped to conduct participatory research and healthcare in tandem with DRR in a location with limited accessibility without comprising safety or ethics.

In contrast, one challenge in such work is planning and preparing for a transition from greater accessibility to more limited accessibility. New Zealand as a country does not typically display limited accessibility for linking DRR and healthcare—instead, it is a world leader in it [129,130]—but the Whakaari 2019 eruption made the country have limited accessibility with respect to mitigating further illness and injury in the specific incident. It would likely be similar after a major Wellington earthquake, depending on the exact damage. Participatory research has been conducted to prepare for a major earthquake in the Wellington area [131], yielding opportunities to prepare and to understand the context of moving from greater accessibility to more limited accessibility due to the earthquake. This work is especially helpful in the context of knowing that participatory research is unlikely to be feasible to a great extent immediately after an earthquake due to the ongoing post-disaster operations. Furthermore, participatory research provides an opportunity to develop bespoke DRR strategies for the people in Wellington, demonstrating person-centered DRR research, which reflects the expected standard of healthcare provision in response to a disaster. Participatory research would empower people who would be in an area of limited accessibility should an earthquake occur, which is important because local people would initially be the responders to a disaster in that location of temporarily limited accessibility.

The 2020 COVID-19 pandemic is another example of limited accessibility emerging. Healthcare systems became overwhelmed with suspected COVID-19 cases as countries entering lockdown limited wider access to healthcare. One consequence was the failure to mitigate further illness and injury for all prospective patients, both COVID-19 and non-COVID-19, who were unable to obtain diagnoses or healthcare, such as numerous cancer patients in the UK [132]. Poor long-term management of the healthcare system in the UK [133] meant that, to deal with COVID-19, the healthcare system had to have restrictions imposed. The UK is rarely defined as a location with limited accessibility, yet the healthcare system had limited accessibility for months as COVID-19 cases peaked and declined.

In the early part of the UK’s first period of lockdown, over 100 health and care workers died from COVID-19 [134], many others were exhausted and experienced adverse mental health impacts, and the system was stretched. While plenty of data were collected and analyses have now started [132,134], conducting participatory research would not have been possible without putting workers and patients at risk. Thus, a significant challenge of participatory research in locations of limited accessibility is illustrated in terms of the lack of resources to gather and analyze perishable data.

Similar situations arose in New York City on 11 September 2001 due to the terrorist attacks, including a hospital’s loss of electricity and communications [135], and in places in Thailand after the 26 December 2004 tsunami [136]. For many not affected directly by the terrorism or tsunami, respectively, healthcare accessibility effectively became limited temporarily for mitigating further illness or injury despite New York City and many of the Thai locations not being remote, isolated, or marginalized. In both disasters, many healthcare workers and patients would not have known whether their families were among the casualties, so participatory research would be of lower priority than keeping themselves and their patients healthy, while checking on people they know.

Combat yields parallel circumstances of induced limited accessibility presenting a participatory research challenge, due to hospitals being bombed in combat zones [137] or terrorist attacks on facilities [138]. In these cases, participatory research might not be safe, irrespective of ethics approval. A further participatory research challenge emerges for combat research, which also applies to terrorism research, that wars or terrorist attacks would be needed in order to carry out the participatory research for determining how to mitigate further illness or injury. This form of “ambulance chasing” for disaster-related research has been critiqued [70] and is known as both a challenge and opportunity for research, which is pre-planned to occur in response to emerging and evolving adverse situations [139].

Consequently, rather than knowing that a location necessarily has limited accessibility, accessibility can increase or decrease swiftly, as further illustrated by the COVID-19 pandemic by locations from Melbourne to Manchester experiencing local lockdown in July 2020. Despite this challenge, an opportunity emerges in that participatory research can assist in preparing people through training, scenarios, exercises, and serious gaming [43,140,141]. This work yields an opportunity to link DRR and healthcare more robustly to cover any directions and rates of changes in a location’s accessibility, while balancing what and who needs to get into a location with suddenly limited accessibility (e.g., aid supplies and first responders) compared to what and who might need to get out (e.g., visitors or people requiring specialized healthcare).

Another challenge of participatory research within the contexts here is recognizing and mitigating the physical or psychological deterioration of patients and of healthcare workers while conducting the research. Numerous methods exist to measure mitigation, including simulation or tabletop exercises [142], modeling [66], framework analysis [143], and integrated risk assessment [144]. This variety of approaches offers opportunities for using multiple methods and then comparing and contrasting the results to hone understanding and to support participants’ creativity and skill development. These opportunities support the desired participatory research without potentially impeding operational work, although the challenge is that people might respond differently in reality than they do in other circumstances.

An opportunity to enact real-time participatory research comes from social media, since postings can be monitored and analyzed as they are happening, followed by post-hoc analysis [145,146]. One advantage is that locations with limited accessibility are easily included in the research without the need for travel, reducing the possibilities for interfering with operations. One disadvantage is that those with access to social media and those willing to post on social media are not necessarily a representative sample of the affected population; in fact, not all locations with limited accessibility even have internet coverage. Disinformation, misinformation, people paid to post (typically from an ideological standpoint or for commercial gain), influencers, boosted posts (paying the social media company to promote the post), and bots all skew the samples and hence the results and findings. The data nonetheless remain representative of what is posted, even if not of what is happening, although explanation would be required to highlight the limitations and advantages of such data. An additional challenge is how much social media postings could be considered to be participatory. Is real-time reporting, videoing, and photographing—including through interactive processes such as streaming—from a disaster scene observation or participation? Defining this perspective and providing a rationale for it is a challenge for participatory research, but once again, transparency enables participatory research to be recognized and accepted in its own right.

One important consideration for skill sets and operational work is the difference between acute and chronic healthcare in locations with limited accessibility. For both acute and chronic healthcare, the healthcare provider requires training to be able to meet the patient’s needs in challenging circumstances [147]. Relying on clinical experience only can increase the risk of human error and the patient experiencing further illness or injury. In contrast, secondary care settings and prehospital care practices are premised on opportunities being available to replenish resources and to medevac or provide other logistical support [148]. Managing long-term conditions or additional care needs in locations with limited accessibility requires DRR strategies, thorough risk assessment, and planning, entailing robust and adequate training [100]. Participatory research could and should be part of such work, providing an opportunity to examine these processes systematically and verifiably for improving training and practice without putting people’s lives at risk. As with social media, challenges regarding the role of participation arises, particularly how participatory research is where the patient cannot or does not participate, even where healthcare professionals are participatory subjects. After all, the aim of mitigating further illness and injury is meant to serve the patient and participatory research is meant to involve those who are being served. The opportunity remains in improving links between healthcare and DRR, which would also increase the safety of healthcare professionals and the quality of their work which, in turn, does indeed serve the patients [149].

Emerging from Section 2 and Section 3, as well as the synthesis and further discussion in this section, Table 4 concatenates the challenges and opportunities identified, indicating specific steps to undertake for participatory research to link DRR and healthcare in locations with limited accessibility.

## 5. Conclusions

This paper has provided a baseline of theory, examples, and analyses to contribute to answering the research question, “What challenges and opportunities occur when participatory research links DRR and healthcare to mitigate further illness and injury in locations with limited accessibility?” Further research would help to delineate more clearly situations where limitations of participatory research cannot or should not be overcome, not just where personnel and resources for research are unavailable due to ongoing operations, but also where collecting data might change decisions being made. The latter is particularly important in case decisions might be changed for the better, which means trying to understand how to identify when improvements would occur through the knowledge that operations are being researched. Where research of real situations is deemed to be infeasible, then more work is needed to determine the design, relevance, and applicability of research using fieldwork with controlled conditions, lab work, and scenarios.

One significant lacuna in this field overall is collecting data about the perspectives and decisions of people who died due to a lack of DRR or healthcare, which also means developing methods to do so as well as to validate results. With the advent of real-time communications and postings through social media during disasters, as discussed in Section 4, words, images, and videos from non-survivors might be extracted, preserved, and analyzed for content about their decision-making and actions, subject to ethics.

Similarly, to ensure that baseline work in both DRR and healthcare informs the new participatory research, the starting point should be DRR and healthcare as preventative processes. Both disasters and health are socially determined and should highlight prevention rather than treatment and cure [10,11,12,16,17,18]. In disaster response and reconstruction, DRR could be better applied [150,151] alongside illness care better adopting healthcare approaches [152]. How to engrain the ethos of prevention as a long-term process in participatory research for mitigating further illness and injury is an ongoing research task, which is part of using participatory research to apply prevention within response, treatment, and cure. Emergency resources can be used, and sometimes are used, to avoid an emergency—such as firefighters advocating for smoke detectors [153] and medical professionals advising which occupational protective equipment to use [154]—but the operational aspects of doing so in locations with limited accessibility remain understudied. The ultimate goal of this participatory research is learning how to connect pre- and post- actions for DRR and healthcare together.

In addition to identifying these areas of further research, this paper has contributed to examining and critically appraising challenges and opportunities for using participatory research for linking DRR and healthcare in locations with limited accessibility. Two main challenges are grounded in identifying and collecting the data, leading to an overarching challenge of potentially divergent goals between research and practice of DRR and healthcare. Participatory research nevertheless provides opportunities for strengthening the association of DRR and healthcare in areas with limited accessibility, but its limitations need to be acknowledged and overcome with transparency.

## Figures and Tables

**Table 1:** Examples of limited accessibility (given no agreed definition).

Situation	Permanent or Temporary Limited Accessibility	Current Example
Armed conflict or war	Temporary	Yemen
Deserts	Permanent	Gobi Desert
Natural hazards	Permanent or temporary	The 2005 Kashmir earthquake, cutting several roads
Islands	Permanent or temporary	St. Helena opened its first airport in 2016, increasing its accessibility
Jungle, forest	Permanent	Amazon villages
Mountains	Permanent	Everest Base Camp
Oceans	Permanent	South Atlantic Ocean
Off-shore	Permanent	Petroleum exploration and extraction rigs
Outer space	Permanent	International Space Station
Polar regions	Permanent	Amundsen-Scott South Pole Station
Terrorism	Temporary	Bombings on London’s public transportation in 2005 shut down the system, limiting people’s options to move in and out of the city

**Table 2:** Categories of data-related challenges.

Defining or Collecting Data?	Challenge
	1. Defining the case study.
Section 2.1	2. Defining the unit of analysis.
	3. Rarity/small sample size.
Defining the data.	4. Uniqueness/comparability.
	5. Transferability/generalizability.
	6. Predictive or explanatory models not robust.
	7. Priority is mitigating further injury or illness rather than research.
	8. Data are not ethically feasible to collect.
Section 2.2	9. Data are not operationally feasible to collect.
	10. The researcher’s presence might influence decisions.
Collecting the data.	11. Individuality of treatment.
	12. Baseline or control conditions might change during the research.

**Table 3:** Categories of data-related challenges for Section 3’s examples.

Example	Predominant Data-Related Challenges from Table 2
Burma snakebite	3, 4, 6, 7, 10
Antarctic auto-appendicectomy	3, 4, 5, 6, 7, 10, 11, 12
Antarctic cancer	3, 4, 5, 6, 7, 10, 11, 12
Antarctic seal	3, 4, 5, 6, 7, 8, 9, 10
Utøya terrorism	5, 6, 7, 8, 9, 10
Whakaari eruption	5, 6, 7, 8, 9, 10, 12
Everest Base Camp avalanche	5, 6, 7, 8, 9, 10, 11, 12
War zones	1, 2, 5, 6, 7, 8, 9, 10, 11, 12

**Table 4:** Recommendations to manage data-related challenges.

	Challenge	Opportunity
	1. Defining the case study.	1. Analyze conceptually the context.
Section 2.1	2. Defining the unit of analysis.	2. Use findings from concept analysis to inform the unit of analysis decision.
Defining the data.	3. Rarity/small sample size.	3. Describe transparently the sample’s representativeness or rarity, declare limitations, and take advantage of apparently unique examples.
	4. Uniqueness/comparability.	4. Describe explicitly the impact and relevance of dissemination.
	5. Transferability/generalizability.	5 and 6. Accept limits of transferability, generalizability, prediction, and explanation while valuing the importance of apparently unique examples.
	6. Predictive or explanatory models not robust.	
	7. Priority is mitigating further injury or illness rather than research.	7. Justify the limits of the research by declaring the priorities and not undertaking unethical research.
	8. Data not ethically feasible to collect.	8. Conduct retrospective, post-hoc analysis.
Section 2.2	9. Data not operationally feasible to collect.	9. Consider alternative methods of data collection, such as post-hoc analysis, scenarios, and field or lab work under controlled conditions.
Collecting the data.	10. The researcher’s presence might influence decisions.	10. Explain clearly the role of the researcher in the participant information and reiterate this when seeking informed consent. Aim to use the researcher’s presence to improve rather than inhibit decisions.
	11. Individuality of treatment.	11. Refer back to fundamental principles of evidence-based practice and identify person-centered approaches.
	12. Baseline or control conditions might change during the research.	12. Document accurately these adaptations and declare them in publications.

## Data Availability

Data sharing is not applicable to this article.
